# Calcium bilirubinate sludge causes early onset of congenital biliary dilatation: a report of two cases

**DOI:** 10.1186/s40792-021-01175-x

**Published:** 2021-04-13

**Authors:** Shoko Kato, Kenitiro Kaneko, Nozomi Matsushita, Shintaro Kurahashi, Takaaki Osawa, Tatsuki Matsumura, Takuya Saito, Yasuyuki Fukami, Shunichiro Komatsu, Tsuyoshi Sano

**Affiliations:** Department of Surgery, Aichi Medical University, 1-1 Yazakokarimata, Ngakute, Aichi 480-1195 Japan

**Keywords:** Congenital biliary dilatation, Choledochal cyst, Obstructive cholangiopathy, Neonatal hemolysis

## Abstract

**Background:**

Symptomatic congenital biliary dilatation (CBD) during early infancy is always characterized by cystic dilation of the common bile duct with a narrow segment connecting the pancreatic duct.

**Case presentation:**

In two consecutive infants with a prenatal diagnosis of CBD, we found that biliary sludge had formed in the cyst upon the appearance of symptoms including acholic stool and hypertransaminasemia. Infrared absorption spectrometry revealed that the sludge consisted of calcium bilirubinate.

**Conclusion:**

We suggest that overproduction of bilirubin by neonatal hemolysis causes sedimentation of bilirubin calcium, resulting in obstruction of the narrow segment and development of symptoms.

## Background

Congenital biliary dilatation (CBD), also known as a choledochal cyst, is a malformation characterized by a dilated common bile duct and pancreaticobiliary maljunction [1]. Children with CBD develop characteristically intermittent symptoms such as abdominal pain, vomiting, jaundice, and elevated levels of serum transaminases and amylase. These symptoms are caused by protein plugs that form in the bile duct because of pancreatobiliary reflux and obstruct the common channel or the narrow segment distal to the dilated bile duct [2, 3]. However, some neonates and young infants with CBD present with obstructive cholangiopathy, which is similar to that seen in biliary atresia [4, 5]. In most such patients, CBD is diagnosed prenatally and is likely to cause liver fibrosis [4]. The symptomatology during early infancy is different from that in older children, but the precise mechanism is unknown. We herein report two cases that may help to illustrate the underlying mechanism.

## Case presentation

### Case 1

**Fig. 1:**
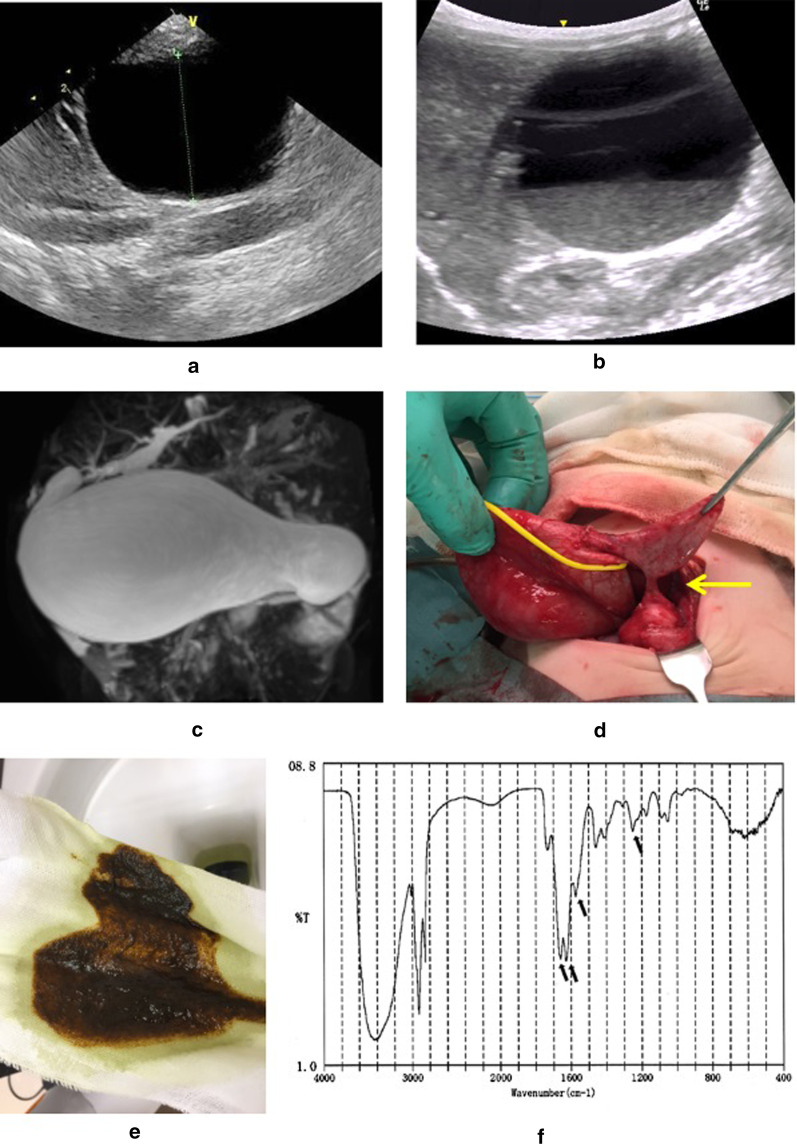
Data of Case 1. **a** Ultrasonography on day 2 showed a large choledochal cyst but no debris. **b** Ultrasonography on day 45 revealed debris in the cyst. **c**, **d** Magnetic resonance imaging and surgical findings confirmed a huge cyst and a narrow segment (arrow). **e**, **f** Infrared absorption spectra of the debris. Four major absorption bands (arrows) were characteristic of calcium bilirubinate

**Fig. 2:**
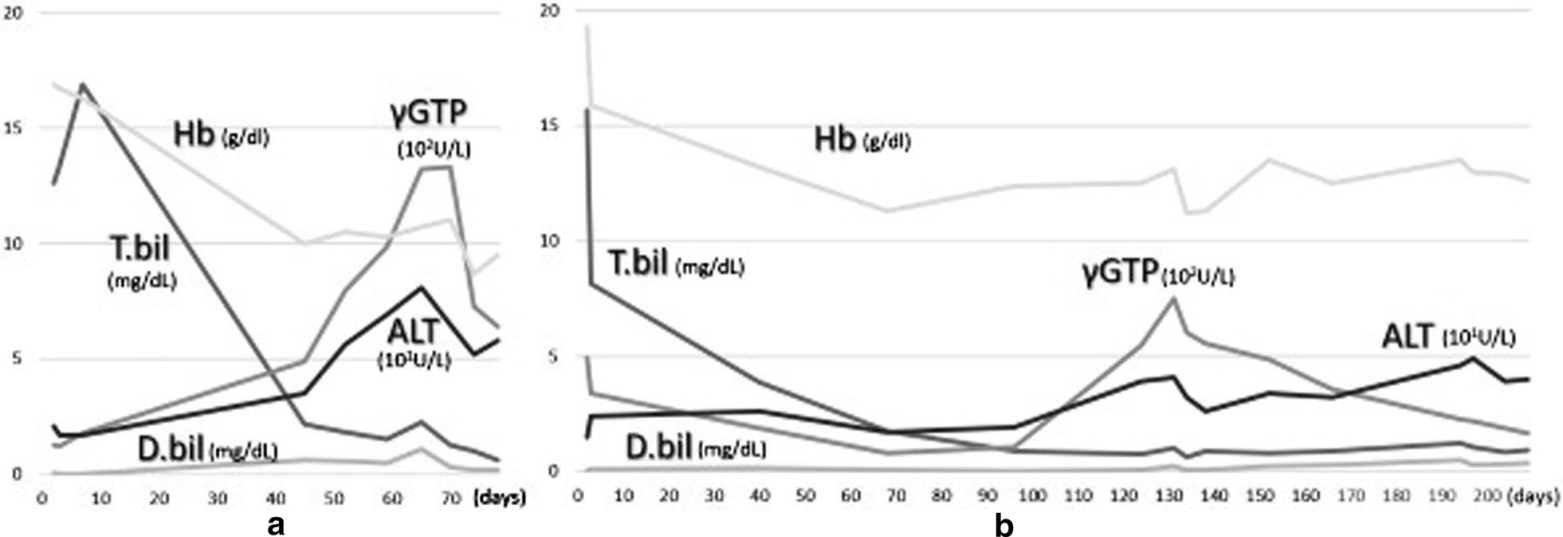
Laboratory data. **a** Preoperative laboratory data of Case 1. The transaminase and gamma-glutamyl transpeptidase (γGTP) concentration were elevated, but the direct bilirubin concentration was not significantly increased. **b** Preoperative laboratory data of Case 2. The hemoglobin level gradually decreased but the transaminase and γGTP concentrations did not increase during the neonatal period. However, the serum levels of transaminases and γGTP began to gradually increase at 4 months

At 27 weeks’ gestation, ultrasonography revealed a cyst in the abdomen. A female neonate was born at 40 weeks’ gestation. A huge choledochal cyst was confirmed by ultrasound and magnetic resonance cholangiopancreatography. The neonate was asymptomatic until she developed acholic stool at 37 days of age. At 45 days, ultrasonography revealed debris in the cyst (Fig. 1b), and laboratory testing showed elevated serum transaminase levels (Fig. 2a). At 72 days, we performed choledochal cyst excision and confirmed a narrow segment distal to the huge cyst (Fig. 1d). The bile contained a large quantity of sludge. Infrared absorption spectrometry revealed that the sludge consisted of calcium bilirubinate (Fig. 1f). The amylase level in the bile was 5 U/L, and the lipase level was 66 U/L (24 and 8 U/L in the serum, respectively). The total bilirubin and direct bilirubin levels in the bile were respectively 6.07 mg/dl and 4.54 mg/dl.

### Case 2

**Fig. 3:**
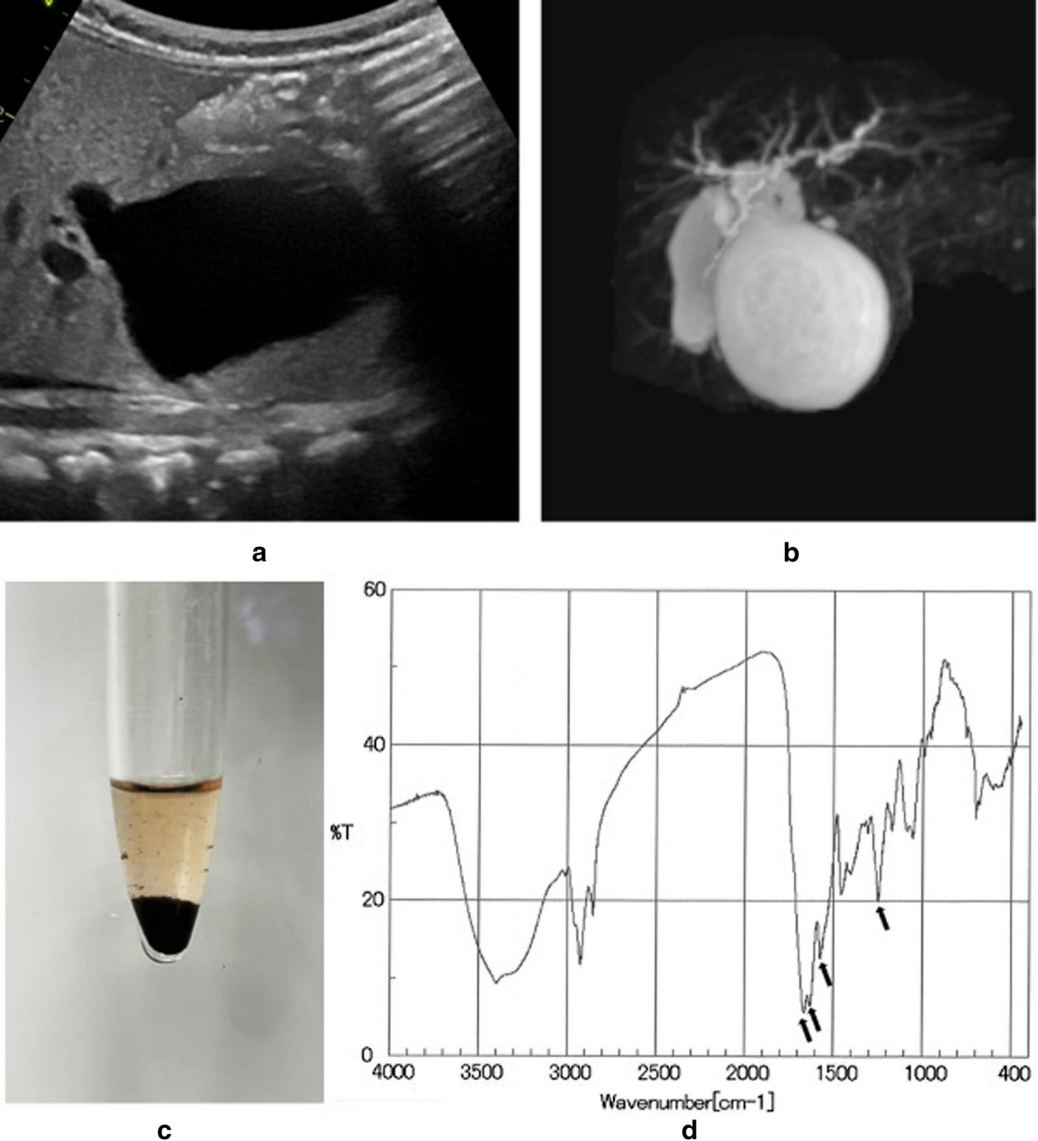
Data of Case 2. **a** Ultrasonography detected debris in the cyst. **b** Magnetic resonance imaging showed a cyst of the common bile duct. **c** The sludge in the bile duct consisted of fine black particles. **d** Infrared absorption spectra of the debris. Four major absorption bands (arrows) were characteristic of calcium bilirubinate

At 29 weeks’ gestation, ultrasonography revealed a cyst in the abdomen. A female neonate was born at 40 weeks’ gestation. A 3-cm-diameter choledochal cyst was confirmed by ultrasound. The neonate was asymptomatic, and her serum transaminase levels were not elevated (Fig. 2b). At 5 days of age, debris appeared in the dilated bile duct (Fig. 3a). At 4 months, her serum transaminase levels began to rise. The cyst gradually enlarged and the diameter was 33 mm at 5 days, 38 mm at 1 month, 44 mm at 3 months, and 50 mm at 6 months. At 6 months, acholic stool appeared and we performed laparoscopic choledochal cyst excision. The narrow segment was too thin to be identified. The bile was yellow and contained sludge (Fig. 3c), and infrared absorption spectrometry showed that the sludge consisted of calcium bilirubinate (Fig. 3d).

The amylase level in the bile was 1 U/L, and the lipase level was 282 U/L (37 and 31 U/L in the serum, respectively). The total bilirubin and direct bilirubin levels in the bile were respectively 14.50 mg/dl and 12.27 mg/dl.

## Conclusions

Symptomatic CBD during early infancy is inevitably of the cystic-dilated type and is accompanied by a narrow segment distal to the cyst [4, 6]. The fusiform-dilated type of CBD does not cause symptoms during this period [6]. In early infancy, hemolysis accelerates because of physiological polycythemia of red blood cells with a short half-life of fetal hemoglobin. The fetal hemoglobin concentration typically decreases from 77% at 1 day to 52% at 6 to 9 weeks [7]. The association between hemolysis and pigment gallstones is well known [8]. Although biliary sludge had been recognized in neonates with CBD [9, 10], the present report is the first to reveal that the sludge was composed of calcium bilirubinate. Considering these facts, the following mechanism is probable. Neonatal hemolysis results in overproduction of bilirubin, causing a high concentration of bilirubin in bile. Stagnation of bilirubin-rich bile in the cystic-dilated bile duct results in sedimentation of calcium bilirubinate. This debris obstructs the narrow segment, causing obstructive cholangiopathy. The mechanism in neonates clearly differs from that in older infants and children, in whom protein plugs or rarely fatty acid calcium stones cause symptoms [1–3, 11].

The occurrence of obstruction depends on the amount of debris and caliber of the narrow segment. Our first patient had a relatively large caliber of the narrow segment but had a large amount of bile sludge, resulting in obstructive symptoms including elevated transaminase levels. However, incomplete obstruction did not cause complete obstructive jaundice (Fig. 2). The second patient had a smaller amount of debris, which caused no symptoms in the neonatal hemolytic period but resulted in gradually more severe obstructive cholangiopathy over a period of several months. This is the first report to suggest the mechanism of symptomatic CBD in neonates and young infants.

We perform operation for prenatally diagnosed CBD soon after the symptoms and signs of the biliary obstruction occur, which were the elevated levels of transaminase and gamma-glutamyl transpeptidase in Case 1 and acholic stool in Case 2. We wait operation unless obstructive symptoms, because congenital biliary stenoses around the hepatic hilum are more easily treated in older infants. Leaving the stenoses causes later hepatolithiasis [12].

## Data Availability

During the course of this research, no data were analyzed, reused, or generated.

## Abbreviation

CBD: Congenital biliary dilatation
